# Waist Circumference is not Superior to Body Mass Index in Predicting Groin Hernia Repair in Either Men or Women

**DOI:** 10.1007/s00268-021-06359-y

**Published:** 2021-11-02

**Authors:** A. Hemberg, A. Montgomery, H. Holmberg, P. Nordin

**Affiliations:** 1 Faculty of Medicine, Department of Surgical and Perioperative Sciences, Umeå University, Umeå, Sweden; 2 Faculty of Medicine, Department of Clinical Sciences, Lund University, Malmö, Sweden; 3 Faculty of Medicine, Department of Public Health and Clinical Medicine, Umeå University, Umeå, Sweden; 4 Helsingborgs Lasarett, KirurgenHelsingborg, Sweden

## Abstract

**Background and aims:**

A high body mass index (BMI) is considered a risk factor for ventral abdominal wall hernias but protective for the development of groin hernias. The reason for this is unclear. The surrounding abdominal fat in obesity might “protect” and limit the passage through the inguinal canal. The aim was to compare two different methods used for obesity registration in groin hernia patients and to investigate the hypothesis of high BMI/low groin hernia risk phenomenon.

**Methods:**

This was a population-based observational study comparing BMI to waist circumference (WC) as well as their correlations to the quantity of groin hernia repair performed in either sex. Two national registers were crosslinked to a large regional register including information on WC.

**Results:**

A larger WC and a higher BMI were associated with a lower risk of having groin hernia repair in both sexes. There was no difference using either WC or BMI as a risk factor for groin hernia repair in either sex. There was no advantage to using body composition based on WC rather than BMI for surgery indication.

**Conclusions:**

Overweight patients, both men and women, have a lower risk of undergoing groin hernia repair regardless of fat distribution. BMI is a well-established method for obesity registration and is recommended in the evaluation of hernia patients.

## Introduction

Body mass index (BMI) is by far the most commonly used indicator of body constitution. However, BMI does not account for body fat distribution. An easier method for use in large population-based cohorts is to measure the waist circumference (WC), which is known to correlate with the amount of abdominal visceral fat [1, 2]. The distribution of body fat around the waist is a known risk factor for cardiovascular disease making these patients riskier in major surgery [3].

A high BMI increases the risk of developing all types of ventral abdominal wall hernias but decreases the risk of developing groin hernias [4–10]. The reason for this somewhat unexpected difference is unclear. Hypotheses include missed diagnoses and unwillingness from providers to diagnose and repair obese patients due to increased risk for complications [5, 7–10]. To the best of our knowledge, WC and its association with groin hernia occurrence has not previously been described. Understanding if WC affects this risk will add valuable information to explain this unexpected difference.

Our aim here was to compare BMI to WC and their respective correlation to groin hernia surgery occurrence in both elective and emergency setting in males and females by crosslinking data from one regional and two national patient registers. Our hypothesis was that high BMI and/or WC prevents inguinal hernia occurrence and thus also prevents subsequent surgery.

## Material and method

This is a Swedish population-based observational study using data from one regional registry—Västerbotten Intervention Program (VIP)—as well as two national registers: the national groin hernia repair registry “Swedish Hernia Registry” (SHR) and groin hernia diagnoses from the National Patient Registry (NPR). Study approval was from the Regional Ethics Board in Umeå (Dnr 2016-93-32Ö and 2015-343-31Ö).

### Registries

VIP is an ongoing population-based health survey that started in 1985 [11]. Since 1992, it covered the entire county of Västerbotten, Sweden. Each year, VIP invites all citizens aged 40, 50, and 60 years old to participate; (30-year-olds were included until 1995 and then enrolled again in 2014 and thereafter). Each year, 6500–7000 clinical examinations are performed including standardized measurements of BMI and WC. BMI was included from the start in 1985 while WC was added from 2003 and onwards. High WC is an important risk factor for several conditions especially cardiovascular. Patients were enrolled since the start of the VIP since BMI could be a risk factor for hernia development on its own. After index examination, each participant was invited every ten years for reexamination; participation ended at age of 60 years. Participation rates were 66–67% [11, 12].

The NPR was founded in 1964 by the National Board of Health and Welfare (NBHW; Swedish: Socialstyrelsen) and includes all inpatient diagnoses coded according to International Classification of Disease (ICD). Participation is mandatory by law. Since 2001, the NPR has also included all outpatient care at surgically specialized clinics. It does not include primary health care visits. Main dropouts were reported are: Personal identification numbers were missing in 1% of cases and the main diagnosis was missing in 1.1% of inpatient cases [13, 14].

SHR was initiated 1992 and covers 97% of all groin hernia repairs performed on patients aged ≥ 15 years in Sweden. Approximately 16 000 operations are registered annually [15]. Patients can be followed over time using their personal identification number regardless of where in Sweden the repair is performed [16]. Hence, a recurrent hernia operation can be traced within the SHR.

### Study population

There were three cohorts:

A. Populations participating in the VIP between 1989 and 2015.
B. Groin hernia diagnosed between 2001 and 2015 identified in NPR.
C. Groin hernia repairs in the county of Västerbotten between 2001 and 2015 identified from the SHR.

Crosslinkage between the three cohorts was performed utilizing the Swedish system via personal identification numbers, which guarantee that each patient was counted only once and that patients could be identified over time regardless of where in Sweden their groin hernia was repaired [16]. Patients with a recurrent groin hernia repair and those with groin hernia repair or diagnosis prior to participation in the VIP were excluded. This was to minimize any possibility of reversed causality. All calculations are patient-based, and a bilateral hernia repair was regarded as a single event.

### Variables and groups

The VIP provided data on BMI, WC, and age. The NPR provided data on the groin hernia diagnose according to the International Classification of Diseases (ICD). The NPR includes both in- and outpatient diagnoses, and the SHR includes surgeries performed as both in- and outpatients. The SHR supplied data on type of hernia, emergent or elective procedures, and date of surgery. Emergent surgery was defined as patients with signs of incarceration repaired within 24 h after arrival. All analyses were stratified by sex due to large differences in groin hernia incidences and in anatomy of the pelvic area [17]. BMI was grouped in four categories. WC was grouped into three categories in accordance with the WHO report [18] (Table 1).

**Table 1:** BMI and WC groups

BMI	
1	<20 kg/m^2^
2	20 to <25 kg/m^2^
3	25 to <30 kg/m^2^
4	≥30 kg/m^2^

Waist circumference	Men	Women
Group A	<94 cm	<80 cm
Group B	94 to <102 cm	80 to <88 cm
Group C	≥102 cm	≥88 cm

### Outcome measures

Primary outcome was the rate of groin hernia repair comparing WC and BMI measures in each sex. Secondary outcomes were differences in frequency of groin hernia being either diagnosed or repaired between sexes with regard to both BMI and WC.

### Statistics

An independent sample t-test was used to compare continuous variables. A chi-square test was used for categorical variables. Missing values were excluded in the analysis. The hazard ratio (HR) and the odds ratio (OR) were calculated using Cox regression and logistic regression, respectively. Confidence intervals (C.I. 95%) are presented using either OR or HR in tables as appropriate.

The plots were created using a logistic regression model. Instead of estimating the effect of BMI as a monotone effect, a restricted cubic spline (RCS) with five knots was used [19]. The RCS values were plotted to illustrate how the effect of BMI and WC varies over the span of BMI and WC without losing the information normally lost in a linear function estimate. The results are presented as a plot with OR and confidence intervals for each individual BMI and WC.

## Results

**Fig. 1:**
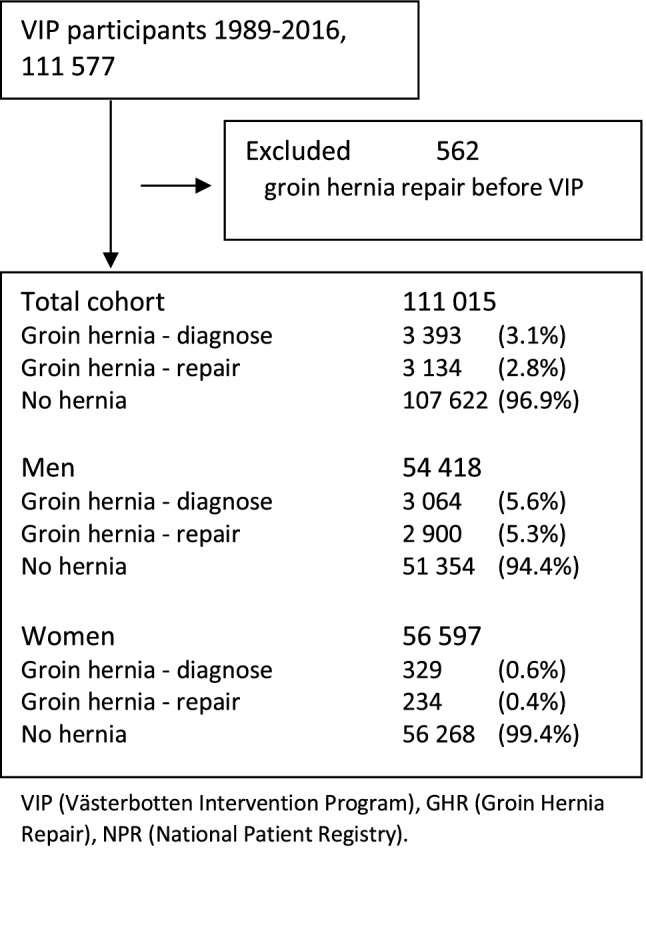
Flowchart of cohort creation

The study was based on 111 577 VIP participants. After exclusion due to a groin hernia repair or hernia diagnosis prior to VIP participation, there were 111 015 subjects eligible for analysis: 54 418 men (49%) and 56 597 women (51%). A total of 3393 (3.1%) out of this cohort were identified from the NPR to have a hernia diagnose and 3134 (2.8%) patients were identified from the SHR as having had a groin hernia repair. The majority had a diagnosed hernia repaired: 80% in men and 61.7% in women (Fig. 1).

The characteristics of the participants are reported in Table 2. A significantly reduced risk of having a groin hernia repaired was seen both in men and women with rising BMI. The same applies to WC. A difference was seen for women in the WC group upon comparing patients with groin hernia diagnosed without having a hernia repair to patients subsequently having had a repair. The mean age at inclusion was significantly higher for repaired versus non-repaired hernias in both men and women. Emergent repairs account for 3% in men and 15% in women.

**Table 2:** Characteristics for non-repaired and non-diagnosed versus repaired and diagnosed groin hernias divided by sex

	No repair (%)	Repair (%)	Total (%)	p	Diagnose, no repair (%)	Diagnose and repair (%)	p
Men (N = 54,418)							
*BMI*							
< 20	874 (1.7)	61 (2.1)	935 (1.7)	< 0.001	7 (1.1)	50 (2)	0.202
20–24.9	18,652 (36.2)	1378 (47.5)	20,030 (36.8)		308 (50.2)	1169 (47.7)	
25–29.9	23,966 (46.5)	1280 (44.1)	25,246 (46.4)		258 (42)	1069 (43.6)	
≥ 30	7740 (15)	153 (5.3)	7893 (14.5)		39 (6.4)	138 (5.6)	
Missing	286 (0.6)	28 (1)	314 (0.6)		2 (0.3)	24 (1)	
*WC*							
Group A*	8127 (15.8)	195 (6.7)	8322 (15.3)	< 0.001	46 (7.5)	193 (7.9)	0.279
Group B*	5505 (10.7)	110 (3.8)	5615 (10.3)		36 (5.9)	110 (4.5)	
Group C*	5745 (11.2)	61 (2.1)	5806 (10.7)		21 (3.4)	61 (2.5)	
Missing	32,141 (62.4)	2534 (87.4)	34,675 (63.7)		511 (83.2)	2086 (85.1)	
*Acute*							
Emergent		86 (3)					
Elective		2814 (97)					
Mean age at inclusion	45.82	49.74		< 0.001	49.43	49.71	0.479
Women (N = 56,597)							
*BMI*							
< 20	3438 (1.7)	18 (7.7)	3456 (6.1)	0.001	7 (5.6)	17 (8.4)	0.086
20–24.9	27,060 (36.2)	130 (55.6)	27,190 (48)		70 (55.6)	112 (55.2)	
25–29.9	17,126 (46.5)	70 (29.9)	17,196 (30.4)		32 (25.4)	59 (29.1)	
≥ 30	8362 (15)	13 (5.6)	8375 (14.8)		17 (13.5)	12 (5.9)	
Missing	377 (0.6)	3 (1.3)	380 (0.7)		0 (0)	3 (1.5)	
*WC*							
Group A*	6693 (15.8)	12 (5.1)	6705 (11.8)	< 0.001	8 (6.3)	12 (5.9)	0.003
Group B*	4924 (10.7)	12 (5.1)	4936 (8.7)		7 (5.6)	12 (5.9)	
Group C*	7406 (11.2)	4 (1.7)	7410 (13.1)		15 (11.9)	4 (2)	
Missing^a^	37,340 (62.4)	206 (88)	37,546 (66.3)		96 (76.2)	175 (86.2)	
*Acute*							
Emergent		35 (15)					
Elective		199 (85)					
Mean age at inclusion	45.92	49.49		< 0.001	48.02	49.66	0.119

Characteristics of 54 418 men from the Västerbotten Intervention Program (VIP) with 2 900 groin hernia repairs added from the Swedish Hernia Registry (SHR) and 56 597 women with 234 groin hernia repairs added from SHR

Group A* with WC of <94 and <80, Group B* 94–101.9 and 80–87.9, Group C* ≥102 cm and ≥88 cm for men and women, respectively. The 3 064 men and 329 women were diagnosed by the National Patient Registry (NPR) and were compared to the groin hernia repairs in the SHR because there are groin hernia repairs in the SHR without diagnosis in the NPR, and thus the numbers do not match. P-values are Pearson’s chi-squared for grouped variables: no repair compared to repair and diagnose as well as no repair compared to diagnose and repair; independent sample *t*-tests were done for mean age at inclusion. ^a^WC measurement started in 2003; missing WC includes missing values as well as all participating before 2003

When comparing the 740 patients with a diagnosed hernia without having had a repair to the 2653 patients having had a repair, a multivariable logistic regression model showed that the risk of having had a surgery was not affected by either BMI or age. “The risk” of having surgery with a diagnosed hernia was lower in females compared to males (OR 0.397, 95% C.I. 0.311–0.507). When using WC instead of BMI, there was a reduced risk for surgery among men with the largest WC (group C; (OR 0.497, 95% C.I. 0.291–0.847)) and in women (OR 0.304, 95% C.I. 0.171–0.540).

Multivariable Cox regression analyses (Table 3) showed a reduction in groin hernia repair frequency with increasing BMI and WC in both men and women; a BMI > 30 in men gave a HR of 0.327 (C.I. 95% 0.277–0.387) and 0.327 in women (C.I. 95% 0.184–0.580).

**Table 3:** Multivariable cox regression analyses for BMI and Waist Circumference for men respective women having had groin hernia repair

	Men			Women		
	HR	95% C.I	p	HR	95% C.I	p
BMI < 20	1.038	0.803–1.341	0.778	1.291	0.788–2.117	0.311
BMI 20–24.9	1.0	Ref		1.0	Ref	
BMI 25–29.9	0.713	0.661–0.770	<0.001	0.756	0.563–1.015	0.062
BMI ≥ 30	0.327	0.277–0.387	<0.001	0.327	0.184–0.580	<0.001
WC Group A*	1.0	Ref		1.0	Ref	
WC Group B*	0.724	0.572–0.915	0.007	1.175	0.525–2.626	0.695
WC Group C*	0.383	0.286–0.511	<0.001	0.238	0.076–0.745	0.014

Multivariate analyses showing the hazard ratio for groin hernia repair separated by sex adjusted for age at inclusion. The BMI analyses was based on 53 500 men with 2 832 groin hernia repairs and 54 937 women from VIP with 226 groin hernia repairs, while the WC analyses are based on 19 578 men with 371 groin hernia repairs and 18 832 women with 28 groin hernia repairs

Hazard ratio (HR) calculated by cox regression analyses adjusted for age at inclusion. *Group A with WC of men and women <94 respective <80, group B 94–101.9 respective 80–87.9, group C ≥ 102 cm respective ≥88 cm for men

**Fig. 2:**
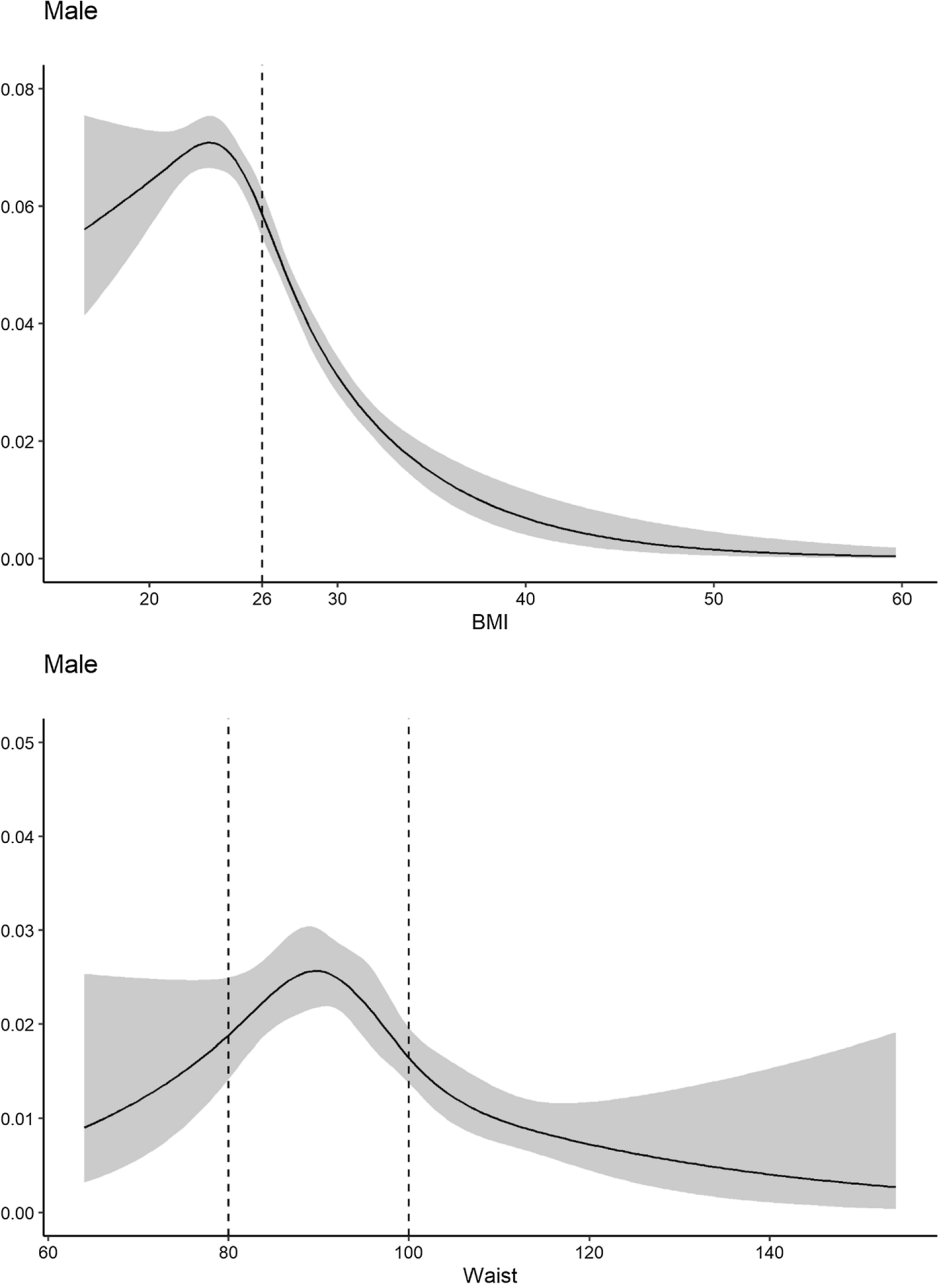
Risk for Groin Hernia Repair plotted for Waist Circumference and Body Mass Index, respectively

**Fig. 3:**
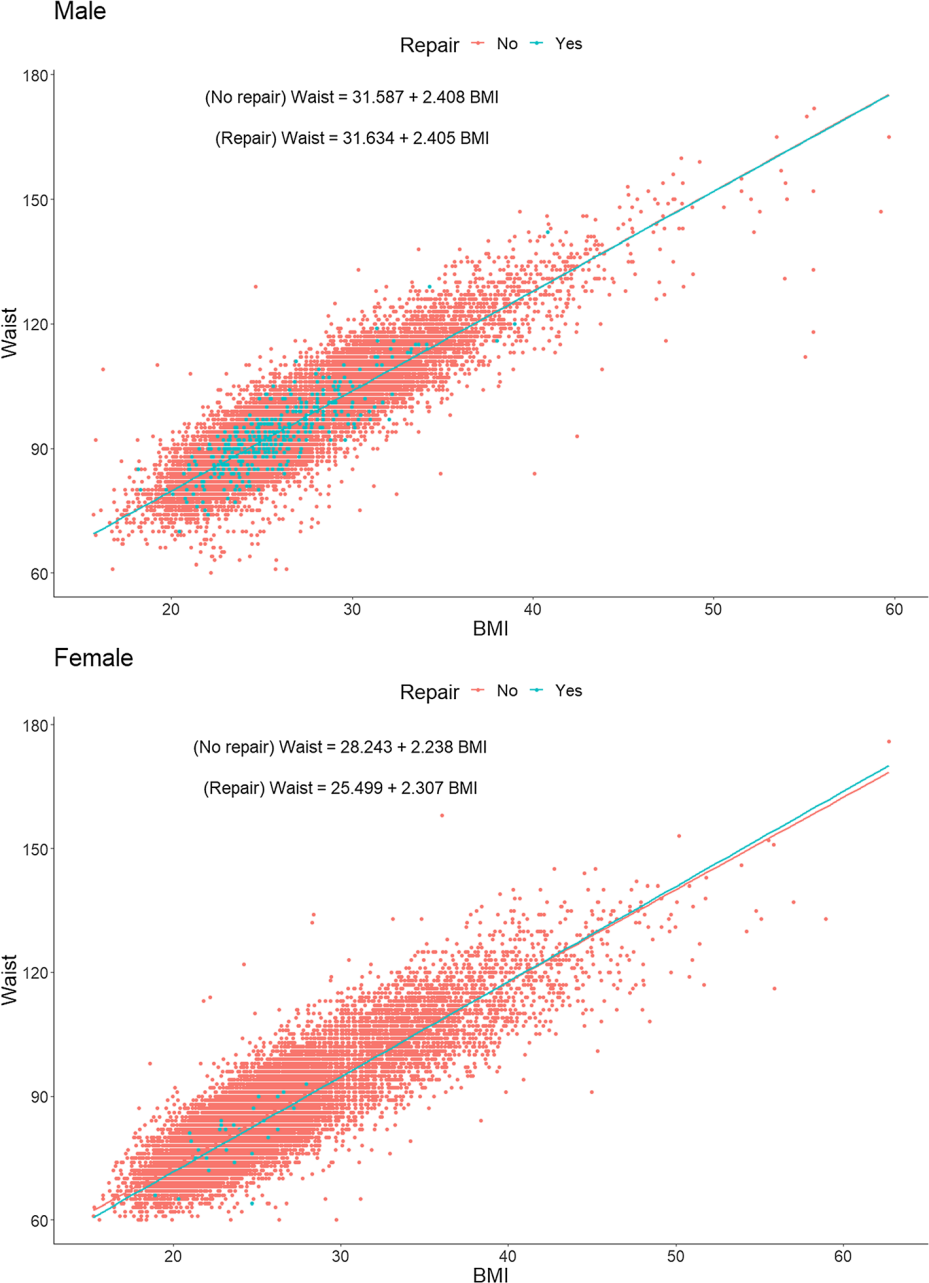
BMI in relation to WC in scatterplots for groin hernia repairs in men and women

The model described above for BMI was used to display the effect of BMI and WC on the risk of having a groin hernia repair (Fig. 2). The WC plot showed an increased risk for males with a WC between 80 and 100 cm while the BMI analysis showed a peak between 21 and 25. This analysis was not performed in women due to an excessively low number of registrations. The relation between BMI and WC with respect to the number of groin hernia repairs is displayed in Fig. 3. There was no difference between BMI and WC as predictors for having a groin hernia repair.

Upon comparing the 121 emergencies to the 3013 elective groin hernia repairs in a multivariable logistic regression model neither BMI nor WC affected the risk of having an emergency repair performed for either sex (Table 4).

**Table 4:** Multivariable logistic regression analyses for emergency repair for men respective women

	Men			Women		
	OR	95% C.I	p	OR	95% C.I	p
BMI < 20	1.679	0.227–12.445	0.957	0.957	0.175–2.607	0.461
BMI 20–24.9	1.000	Ref	0.612	0.612	Ref	0.568
BMI 25–29.9	0.988	0.626–1.559	0.959	0.959	0.355–1.953	0.675
BMI ≥ 30	0.901	0.349–2.327	0.830	0.830	0.095–1.3	0.117

Multivariate analyses showing the odds ratio for emergent groin hernia repair separated by sex adjusted for age at inclusion. The analyses were based on 2872 men and 231 women

Odds ratio (OR) calculated by logistic regression analyses adjusted for age at inclusion

## Discussion

BMI and WC measurements demonstrate different aspects of body fat distribution that might interfere with groin hernia occurrence. It is important to determine if body fat distribution would result in a difference in having groin hernia surgery performed or not. The present risk analysis on fat distribution used a large population-based cohort and showed that there is no difference between fat distribution groups relative to the need for a groin hernia repair; this was seen in both men and women.

The risk to undergo groin hernia repair decreases substantially with BMI > 30 and with a WC > 102 cm for men and >88 cm for women. The risk is higher for those closer to the normal range of WC and BMI than in those markedly above. The relationship for low BMI (<20) and WC (<94 cm for men and 80 cm for women) is more uncertain. No clear conclusions can be drawn from this study due to a lack of participants.

The strengths of this study include the large number of participants overall as well as the fact that the study used well-established and well-validated national registers with high coverage of both the SHR and the NPR for diagnoses and surgery data. To mitigate the risk of using potentially biased variables, hernia patients either diagnosed or repaired were compared to two different variables for body composition and obesity.

VIP participation was voluntary with 66% accepting. This is a comparatively high number joining a voluntary register. There might be a potential risk for selection bias. A previous VIP study showed almost 10% more low-income and single individuals among those not participating [12]. A small difference of <5% was also seen with regard to education, age, urbanization, and country of birth. VIP is an interventional program including a planned follow-up with health advice. It is based on the participant’s results, and this feature could positively affect participation. Weight analysis showed that two-thirds of the VIP participants gained weight between the examinations with a mean weight change of 5.5% for men and 6.6% for women [20]. VIP began measuring WC in 2003 and reduced the number of WC values available for analysis. The incidence of groin hernias among females is generally low; thus, there were too few participants for robust conclusions.

Previous research has shown a positive correlation between high BMI and ventral hernias [4]. The opposite is shown with a lower risk for groin hernia repair in individuals with a high BMI [5–10, 21]. This study confirmed the lower risk for both high BMI and WC. The risk for groin hernia repair seems to be more common among those having a normal BMI and WC than those with higher BMI values. The relationship between the groups with the lowest BMI and WC and the risk of groin hernia repair remains uncertain. The confidence intervals are too wide to draw any firm conclusions due to the low number of participants in these groups. Of course, both the volume and distribution of the fat tissue can influence the measurement of the waist circumference. The distribution of fatty tissue in females and males differs substantially. WC is however better correlated to visceral fat than BMI [2].

The differences between groin hernias and other type of hernias with respect to BMI could be because it is easier to detect a groin hernia in patients with a low BMI [5, 7–10]. Obese patients might have more comorbidities and an increased risk for complications making them less suitable for elective repair, thus resulting in surgeons being “unwilling” to surgically treat overweight patients with limited symptoms [8]. This study examined the problem by all available means: using only diagnosed hernias, comparing non-repaired to repaired hernias, elective to emergent repairs, BMI differences, and body fat distribution. In the comparison of patients with a diagnosed hernia with or without repair, the largest WC group had a lower OR for being repaired. This correlation was not seen when using BMI. An “unwillingness to repair” obese patients would have resulted in a correlation to both WC and BMI. The reason for this discrepancy is still unknown. The perplexingly high BMI or WC and low risk for groin hernia phenomenon is not due to an unwillingness to repair nor is it due to missed diagnosis. This would increase the number of emergent repairs. This study cannot explain the underlying cause but does suggest that further research investigates the extra amount of fat tissue in subjects with a high WC and BMI. This fat might provide structural support in the groin area covering the potential narrow and weak areas. This hypothesis correlates well with earlier research showing an increased risk for femoral hernias among women with low BMI were small defects are free from fat coverage and could easily to be entered by organs/tissue when straining [5].

Female sex showed a lower risk for having surgery performed when diagnosed, which is inconsistent with guidelines [21]. However, our study was not designed to evaluate causality. This potential correlation strongly calls for further research and a discussion among surgeons.

To the best of knowledge, associations between WC and groin hernia have not yet been studied whereas BMI has [4–10, 17]. WC is probably a more accurate measure than BMI in demonstrating the local abdominal fat distribution including visceral fat depots [1, 2]. BMI and WC follow each other closely when analyzed using a scatterplot. No measure seems to be superior to the other.

## Conclusion

The risk of having a groin hernia repair is lower for those with a BMI or waist circumference (WC) above average. There is no benefit to differentiating body composition via WC versus BMI for indication of surgery. BMI is well-established and is recommended for use in the evaluation of hernia patients.
